# Attentional attenuation (rather than attentional boost) through task switching leads to a selective long-term memory decline

**DOI:** 10.3389/fpsyg.2022.1027871

**Published:** 2022-10-20

**Authors:** Michèle C. Muhmenthaler, Beat Meier

**Affiliations:** Institute of Psychology, University of Bern, Bern, Switzerland

**Keywords:** task switching, recollection, remember/know paradigm, attention attenuation, delayed memory

## Abstract

Allocating attention determines what we remember later. Attentional demands vary in a task-switching paradigm, with greater demands for switch than for repeat trials. This also results in lower subsequent memory performance for switch compared to repeat trials. The main goal of the present study was to investigate the consequences of task switching after a long study-test interval and to examine the contributions of the two memory components, recollection and familiarity. In the study phase, the participants performed a task-switching procedure in which they had to switch between two classifications tasks with pictures. After a short vs. a long study-test interval of a week, the participants performed a surprise memory test for the pictures and gave remember/know judgements. The results showed that recognition memory declined after 1 week and this was mainly due to a decrease in “remember” responses. The results also showed that the task-switching effect on memory was enduring. Whereas the results of the immediate test were mixed, the results of the delayed tests showed that the task-switching effect was based on recollection, expressed in more “remember” responses for repeat than for switch trials. As recollection is more sensitive to attention manipulations than familiarity, the results align with the notion that attentional requirements at study determine what we remember, in particular after a long study-test interval.

## Introduction

Attention and memory are fundamentally connected (Chun and Johnson, 2011). While our memories influence what we attend to, attention determines what we remember later (Becker and Rasmussen, 2008). Attending to or focusing on an event enhances the likelihood of encoding this event for later memory retrieval (Chun and Turk-Browne, 2007). For example, transient increases in attention to one task can enhance memory performance in a second task, an effect known as the *attentional boost effect* (Swallow and Jiang, 2010, 2013). In contrast, when cognitive control demands increase, the ability to attend to an item is reduced and as a consequence memory performance is impaired (Gardiner and Parkin, 1990; Lavie, 2010; Richter and Yeung, 2012, 2015; Craik et al., 2018; Muhmenthaler and Meier, 2019; Greene and Naveh-Benjamin, 2022). For example, when people perform multiple tasks simultaneously (Craik et al., 1996; Naveh-Benjamin et al., 1998), when they are distracted by irrelevant stimuli (Jenkins et al., 2005; Wais et al., 2010) or when they have to switch between two competing tasks, target memory is impaired (Richter and Yeung, 2012; Muhmenthaler and Meier, 2019; Dubravac and Meier, 2020; Muhmenthaler and Meier, 2021a). In the present study, we used a task-switching procedure with pictures to manipulate the attentional demands, then we assessed recognition memory either immediately or after a week. The main goal of our study was to extend the research on task switching by investigating recognition performance after a long retention interval. Moreover, we examined the contributions of recollection and familiarity on memory performance. As recollection relies on attention during encoding (Jacoby et al., 1989; Gardiner and Parkin, 1990), we assumed that the effect may be based mainly on recollection.

The task-switching paradigm has been developed as an experimental approach to explore the mechanisms of cognitive control by comparing task switch and task repetition trials (Rogers and Monsell, 1995; Wylie and Allport, 2000). Task switching usually results in slower performance for switch compared to repeat trials (i.e., switch costs) due to the enhanced attentional requirements. Recently, several studies have investigated the impact of task switching on subsequent memory. For example in a study by Richter and Yeung (2012), compound stimuli which consisted of picture–word pairs were used at study and participants had to switch between classifying pictures versus words, after a brief delay recognition memory was tested. The results showed that task switching compared to task repetition resulted in lower recognition memory of the targets. Switching requires more attention than repeating and thus reduces the working memory resources available for encoding these stimuli. The consequence is reduced memory performance (Lavie, 2005; Chun and Turk-Browne, 2007; Uncapher and Wagner, 2009; Chun and Johnson, 2011; Meier and Muhmenthaler, 2021). Several studies have replicated that task switching results in a memory cost for switch stimuli (Richter and Yeung, 2015; Muhmenthaler and Meier, 2019; Dubravac and Meier, 2020). The goal of the present study is to expand this research by investigating the impact of task switching on memory after a longer delay (i.e., 1 week) and to investigate the contributions of recollection and familiarity to the task-switching effect.

Recollection and familiarity reflect two distinct processes of declarative memory (Yonelinas, 2002). Recollection reflects controlled processing and strategic elaboration and is accompanied by vivid and rich contextual details of previously experienced events. Familiarity reflects automatic processing and is accompanied by the feeling that an event has been experienced before, in the absence of contextual information about that event (Jacoby and Witherspoon, 1982; Jacoby et al., 1989; Yonelinas, 2002). The subjective qualitative estimates of memories can be derived using the “remember/know” paradigm which was developed by Tulving (1985). A “remember” response indicates that seeing the stimulus brings back to mind some specific recollection with contextual details of what was experienced. A “know” response indicates that seeing the stimulus brings to mind a feeling of familiarity, without any contextual details (Gardiner and Java, 1991; Hockley and Consoli, 1999).

Dual-process theories posit that both forms of recognition memory decrease with time, but at different rates with a faster decline for recollection than for familiarity (Gardiner and Java, 1991; Hockley and Consoli, 1999; Joordens and Hockley, 2000; Meier et al., 2013). The different trajectories of the memory components provide evidence that they reflect different processes and not just correspond to strong and weak memory traces (Gardiner, 1988; Gardiner and Java, 1991). Specifically, the processes underlying recollection are more attention demanding than familiarity-based processes. This is reflected in the evidence that full-attention conditions at study lead to more “remember” responses than divided-attention conditions, whereas “know” responses are quite unaffected (Jacoby and Witherspoon, 1982; Gardiner and Parkin, 1990; Mangels et al., 2001; Yonelinas, 2002). As the task-switching effect also relies on attentional processes, it is straightforward to assume that it is associated with recollection. Indeed in a recent study, the task-switching effect was driven by significantly more “remember” responses in repeat than in switch trials, whereas “know” responses did not vary with task switching (Muhmenthaler and Meier, 2019). However, it is unclear whether this effect is enduring.

### The present study

In two task-switching experiments, we used pictures as stimuli and the participants had do classify them as “smaller” or “bigger than a soccer ball” or as “living” or “non-living*”* (*cf.*, Muhmenthaler and Meier, 2019). All the stimuli could be used for both tasks, the stimuli were therefore bivalent (Woodward et al., 2003). After the study phase, a surprise recognition memory test was conducted. The participants had do decide whether a stimulus was “old” or “new,” then we applied the “remember/know” procedure to assess the estimates of recollection and familiarity (Tulving, 1985; Yonelinas, 2002). In Experiment 1, the participants performed the recognition test either immediately or after 1 week. In Experiment 2, in order to increase the statistical power for the delayed task switching on memory effect, all the participants performed the memory test after 1 week.

We expected overall lower recognition memory performance after 1 week. As recollection-based memory declines more rapidly than familiarity-based memory, we hypothesized that the decline would be due to a decrease in “remember” responses (Gardiner, 1988; Gardiner and Java, 1991; Sadeh et al., 2014). Based on previous research, we expected a task-switching effect on immediate memory. We further hypothesized that this effect may be enduring and thus be intact in the delayed tests. Due to more available attentional resources in repeat than in switch trials, more elaborated processing is possible, and this boosts sustainable learning, that is, a benefit after a longer delay (Gardiner, 1988; Gardiner and Java, 1991; Meier and Muhmenthaler, 2021). As recollection is more sensitive to attention manipulations than familiarity, we expected that the task-switching effect would be based on recollection. We wanted to explore whether this effect would be enduring.

## Experiment 1

### Method

#### Participants

The participants were 80 undergraduate students (18 male and 62 female) from the University of Bern. The age ranged from 18 to 31 years (*M* = 22.10, *SD* = 2.47) and they received course credits for participation. Due to the pandemic, the experiment was conducted as an online-study. The study was approved by the local ethical committee.

#### Material

For the experimental trials, a total of 128 colored photographs were used (*cf.* Muhmenthaler and Meier, 2019). The stimuli derived from four categories: Objects which were larger than a soccer ball and living (e.g., an elephant), larger than a soccer ball and not living (e.g., a car), smaller than a soccer ball and living (e.g., a fly), smaller than a soccer ball and not living (e.g., a lipstick). All the stimuli could unambiguously be classified both as smaller-or-bigger than a soccer ball and as living or non-living, thus the stimuli could be used for both tasks, that is, they were bivalent. Each stimulus category involved 32 stimuli. Stimuli were arranged in separate lists of 64 pictures, counterbalanced across trial type, classification task and assigned response key. One of the lists was used in the study phase, and both lists were presented in the test phase. Lists were counterbalanced across participants. Four additional stimuli were used for a short practice block, one per category.

The experimental task was programmed with the Open Sesame interface (Mathôt et al., 2012). The study was hosted on a JATOS open-source server (Lange et al., 2015).

#### Procedure

After signing up for the experiment, the participants received web links *via* email. Half of the participants were assigned to the immediate test condition. They received three web links with the instruction to open them in a given order. The first link contained the study phase, the second link contained a questionnaire about digital habits (to create a filled retention interval of about 10 min) and the third web link contained the test phase. The other half of participants were assigned to the delayed test condition. They received the study phase link only. They were told that they would receive a second link 6 days later with the instruction to perform the second part of the experiment exactly at the same day and time 1 week after they conducted the first part. They were not informed that their memory would be tested a week later.

##### Study phase

**Figure 1:**
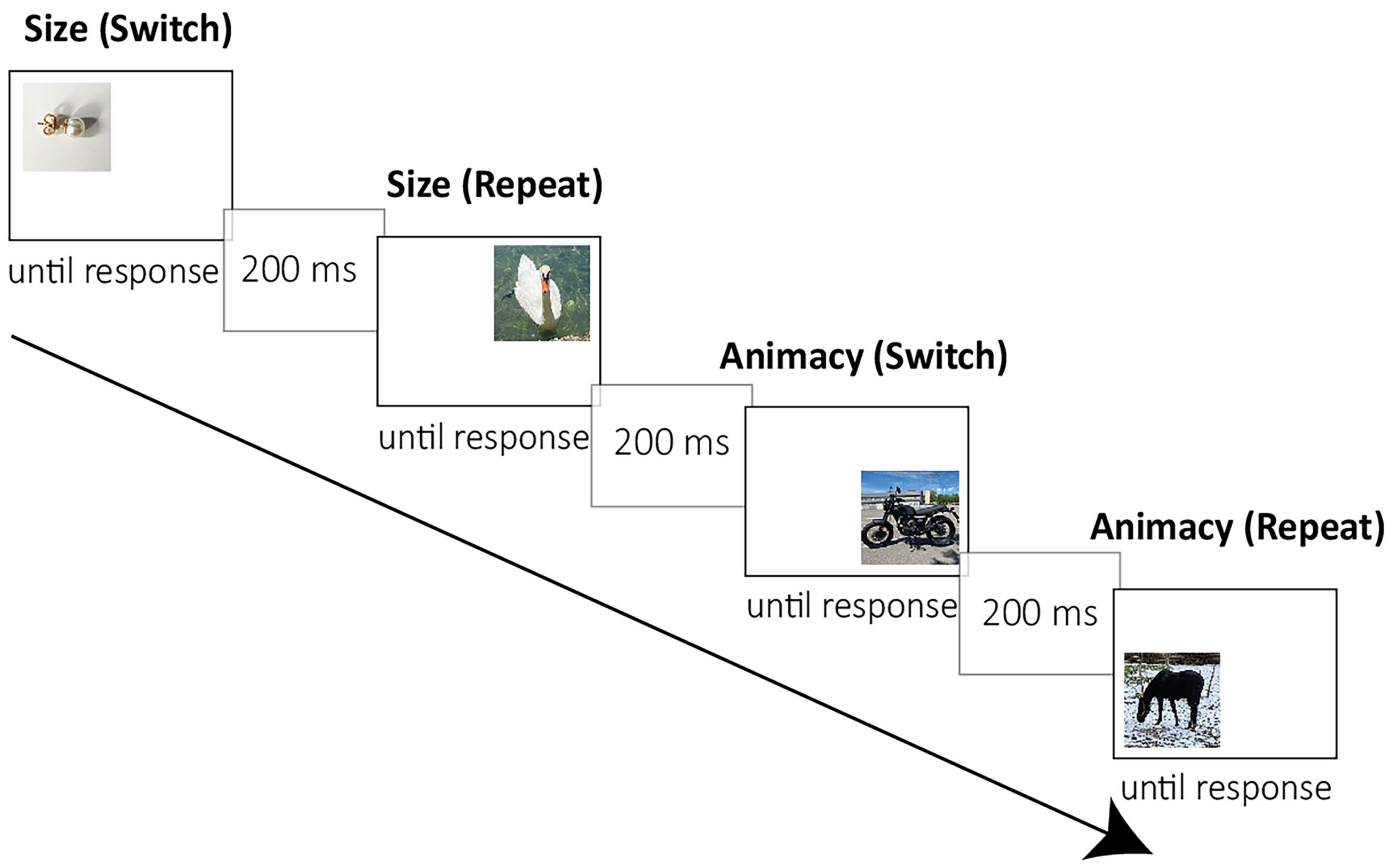
Example of a study trial sequence. The participants had to switch between two tasks in a predictable AABB order. All the stimuli were bivalent (that is, they could be used for both tasks). All the images in the figure are photographs taken by MM.

In the study phase, the participants were instructed to categorize stimuli as fast and as accurately as possible and to switch between the two tasks in a predictable AABB order. Participants had to perform the size task (smaller or bigger than a soccer ball) when the stimulus appeared in the upper part of the screen, and to perform the animacy task when it appeared in the lower part. The stimuli were presented clockwise, beginning in the upper half, see Figure 1. Half of the participants had to press the *a*-key when an object was bigger than a soccer ball or living, and the *l*-key when the object was smaller than a soccer ball or non-living. For the other participants, the response key assignment was *vice-versa*. The stimuli were presented until a response key was pressed, then the next stimulus was presented after a response–stimulus interval of 200 ms. The stimuli were presented randomized, each task twice in succession. After a brief practice phase with eight trials, participants performed the study phase with 64 trials.

##### Test phase

The test phase involved a surprise recognition memory test. Half of the participants conducted the test phase after a short retention interval, the other half conducted the test phase after 1 week. They were informed that they would see more pictures and that they had to indicate whether they had seen each picture already during the first phase of the experiment by pressing the j–key for an old-response or by pressing the n-key for a new response. In case of an old-response, they were asked to give an additional “remember/know” judgement by pressing the 1-key for “remember” or the 2-key for “know” on the number pad. They were instructed to give a “remember” response when they were sure that they had seen the picture and to give a “know” response when they perceived a feeling of familiarity. For each trial, the stimulus was presented until a response key was pressed. The stimuli appeared in randomized order with a response stimulus interval of 200 ms. One half of the stimuli were old (presented in the study phase) and the other half were new (not presented in the study phase).

#### Statistical analyses

In an *a priori* power analyses we computed the sample size as a function of the required power level, the significance level and the population effect size we expected, using G*Power for dependent-samples t-tests (Faul et al., 2007). We used an expected effect size for task switching of *f* = 0.25, based on previous results (*cf.* Muhmenthaler and Meier, 2019), a significance level of 0.05 and 0.90 as power level. The analysis computed 36 participants as an optimal sample size. As we conducted two independent recognition memory tests and due to counterbalancing considerations, we decided to test 80 participants (40 per test).

For the study phase, task-switching performance was analyzed using two-tailed paired sample *t*-tests for reaction times (RTs) and accuracy. For the test phase, we conducted a 2 × 2 ANOVA on study-test interval and on task switching, followed by planned paired sample *t-*tests on the task-switching on memory effect, separately for the immediate and the delayed test. As it is not possible to assign the false alarm rates to repeat or switch trials, we used hit rates only as recognition scores (Ortiz-Tudela et al., 2017; Muhmenthaler and Meier, 2019). To assess the contribution of recollection and familiarity on memory performance, “remember” and “know” responses were analyzed accordingly. An alpha level of 0.05 was used. Effect sizes are expressed as Cohen’s *d*. Non- significant results were followed up by Bayesian analyses.

### Results

#### Study phase

Task switching performance was analyzed using two-tailed paired sample *t*-tests for accuracy and RTs. We considered responses slower than 200 ms and longer than 2,500 ms as outliers (11.9% of all responses). The analysis of the accuracy revealed that participants were more accurate on repeat (*M* = 0.94, *SE* < 0.01) than on switch trials (*M* = 0.92, *SE* < 0.01), *t*(79) = 3.48, *p* < 0.001, *d* = 0.389. For the RTs, we moreover excluded error trials (7.7% of all responses). The analysis of the trimmed reaction times revealed faster RT on repeat (*M* = 1165 ms, *SE* = 24) than on switch trials (*M* = 1777 ms, *SE* = 48), *t*(79) = 16.7, *p* < 0.001, *d* = 1.86. The results showed the expected switch costs.

#### Test phase

##### Hits

The 2 × 2 repeated measures ANOVA with the between-subject factor study-test interval and the within-subject factor task switching on the hits (correctly recognized old pictures) revealed better memory performance in the immediate (*M* = 0.80, *SE* = 0.03) than in the delayed memory test (*M* = 0.45, *SE* = 0.03), *F*(1, 78) = 93.50, *p* < 0.001, $\eta_{p}^{2}$ = 0.55. Overall, more repeat stimuli (*M* = 0.64, *SE* = 0.02) were correctly recognized than switch stimuli (*M* = 0.61, *SE* = 0.02), *F*(1, 78) = 8.73, *p* = 0.004, $\eta_{p}^{2}$ = 0.10. The interaction between study-test interval and task switching was not significant, *F*(1, 78) < 1, *p* = 0.844, $\eta_{p}^{2}$ < 0.01. We further conducted planned contrasts for each study-test interval separately. These two-sided paired sample *t*-tests revealed that the task-switching effect on memory remained intact in both tests (immediate: *t*(39) = 2.63, *p* = 0.009, *d* = 0.428; delayed: *t*(39) = 2.07, *p* = 0.045, *d* = 0.319).

##### “Remember” responses

The same 2 × 2 ANOVA on “remember” responses revealed that the study-test interval was significant with more “remember” responses in the immediate (*M* = 0.67, *SE* = 0.02) than in the delayed test (*M* = 0.21, *SE* = 0.02), *F*(1, 78) = 180.74, *p* < 0.001, η_p_^2^ = 70. More “remember” responses were associated with repeat (*M* = 0.47, *SE* = 0.02) than with switch trials (*M* = 0.41, *SE* = 0.02), *F*(1, 78) = 25.12, *p* < 0.01, η_p_^2^ = 24. The interaction between interval and task switching was not significant, *F*(1, 78) < 1, *p* = 0.403, $\eta_{p}^{2}$ = 0.01. We further conducted planned contrasts for both study-test intervals separately. These two-sided paired sample *t*-tests revealed that the effect on “remember” responses on task switching was significant in both tests (immediate: *t*(39) = 3.93, *p* < 0.001, *d* = 0.614; delayed: *t*(40) = 3.04, *p* = 0.004, *d* = 0.469).

##### “Know” responses

The 2 × 2 ANOVA on “know” responses revealed that the study-test interval was significant with more “know” responses in the delayed (*M* = 0.24, *SE* = 0.01) than in the immediate test (*M* = 0.13, *SE* = 0.01), *F*(1, 78) = 29.91, *p* < 0.001, η_p_^2^ = 0.28. More “know” responses were associated with switch (*M* = 0.20, *SE* = 0.01) than with repeat trials (*M* = 0.17, *SE* = 0.01), *F*(1, 78) = 8.19, *p* = 0.005, η_p_^2^ = 0.10. The interaction between interval and task switching was not significant, *F*(1, 78) = 1.56, *p* = 0.215, $\eta_{p}^{2}$ = 0.02. We further conducted planned contrasts for both study-test intervals separately. These two-sided paired sample *t*-tests revealed a significant result in the immediate test (*t*(39) = 2.65, *p* = 0.012, *d* = 0.413) and a non-significant result in the delayed test (*t*(39) < 1, *p* = 0.416, *d* = 0.128). In order to test the robustness of the null effect, a Bayesian analysis was conducted (Dienes et al., 2018). Using JAMOVI, we calculated a Bayesian two-sided paired sample *t*-test on the “know” responses. The resulting BF of 0.313 indicates evidence for the null hypothesis (i.e., is 3 times more likely than the alternative hypothesis; Jarosz and Wiley, 2014). Accordingly, the contribution of “know” responses did not differ for repeat and switch trials in the delayed test.

##### False alarms

In the immediate test false alarm rate was 7% in the immediate test and 27% in the delayed test, *t*(78) = 10.50, *p* < 0.001, *d* = 2.34.

##### Achieved power

The achieved power in the immediate test was 0.84, when calculating power with the empirical effect size of *d* = 0.428, an alpha level of 0.05 and the sample size of 40. The achieved power in the delayed test was 0.63, when calculating power with the empirical effect size of *d* = 0.319, an alpha level of 0.05 and the sample size of 40.

### Discussion

The results replicated that task switching hurts memory for switch stimuli. Moreover, they showed that this effect was enduring. The results also showed more “remember” responses in repeat than in switch trials for both study-test intervals. Thus, the task-switching effect on memory was mainly based on recollection. The results for the” know” responses were somewhat less clear. Overall, familiarity also seemed to contribute to the task switching effect on memory, but planned comparisons revealed a contribution only in the immediate, but not in the delayed test. This may indicate that this contribution washed out over time.

In Experiment 2, we wanted to replicate the delayed memory effects with higher statistical power. Toward this goal, we designed a similar experiment, but all the participants were tested after 1 week only. Moreover, this study was conducted in the lab, thus providing the opportunity to replicate the results of the online study under controlled laboratory conditions.

## Experiment 2

### Method

#### Participants

The participants were 82 undergraduate students (20 male and 62 female) from the University of Bern. The age ranged from 18 to 42 years (*M* = 23.20, *SD* = 3.50) and they participated in the study for course credits. The study was approved by the local ethical committee and all the participants gave their written consent.

#### Materials and procedure

The materials and the procedure were identical to Experiment 1, with the following exceptions: The participants were tested individually in a lab at the University of Bern. The experiment was programmed with Eprime 2.0. Recognition memory was tested only after 1 week, in order to enhance statistical power and as we were mainly interested in delayed memory performance.

#### Analyses

In an *a priori* power analyses we computed the sample size as a function of the required power level, the significance level and the population effect size which we expected, using G*Power for dependent-samples *t*-tests (Faul et al., 2007). We used an expected effect size for task switching of *d* = 0.319, based on the result of the delayed test of Experiment 1, a significance level of 0.05 and 0.90 as power level. The analysis computed 86 participants as an optimal sample size. The statistical analyses were similar to Experiment 1.

### Results

#### Study phase

We excluded 15.8% of all trials (error trials and outliers). The analysis of the trimmed reaction times revealed that the participants were significantly faster on repeat (*M* = 1,203 ms, *SE* = 31) than on switch trials (*M* = 1,663 ms, *SE* = 39), *t*(81) = 18.17, *p* < 0.001, *d* = 2.01. The analysis of the accuracy revealed that participants were more accurate on repeat (*M* = 0.94, *SE* < 0.01) than on switch trials (*M* = 0.91, *SE* < 0.01), *t*(81) = 3.58, *p* < 0.001, *d* = 0.395. The results showed the expected switch costs.

#### Test phase

**Figure 2:**
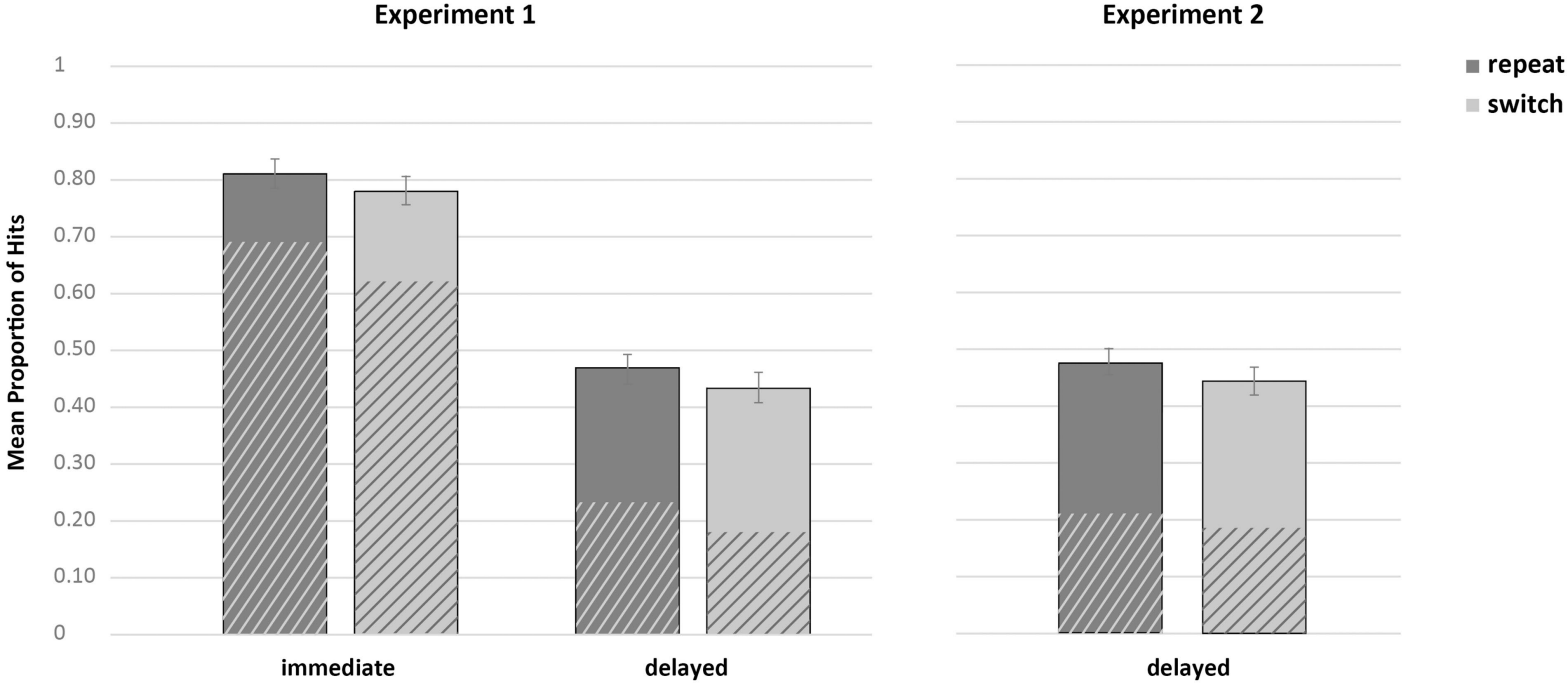
Results of Experiment 1 (left) and Experiment 2 (right), depicted as Hits, separated by remember (shaded area) and know judgements (solid area). The error bars represent standard errors.

The overall recognition memory performance was 46% (*SD* = 0.15) with a false alarm rate of 18% (*SD* = 11). The critical results are depicted in Figure 2. The two-tailed paired sample *t*-test on the hits revealed that more repeat stimuli (*M* = 0.48, *SE* = 0.02) were recognized than switch stimuli (*M* = 0.44, *SE* = 0.02), *t*(81) = 3.04, *p* = 0.003, *d* = 0.335. To assess the contribution of recollection and familiarity on memory performance, additional *t*-tests on “remember” and “know” responses were conducted. The results showed that significantly more «remember» responses were given for repeat (*M* = 0.21, *SE* = 0.01) than for switch trials (*M* = 0.19, *SE* = 0.01), *t*(81) = 2.54, *p* = 0.013, *d* = 0.280. The «know» responses did not vary with trial type (both: *M* = 0.26, *SE* = 0.01), *t*(81) < 1, *p* = 0.714, *d* = 0.041. A Bayesian two-sided paired sample *t*-test on the “know” responses gave a BF of 0.130, indicating that the evidence for the null hypothesis is 8 times more likely than the alternative hypothesis (Jarosz and Wiley, 2014). Accordingly, the contribution of “know” responses for repeat and switch trials did not differ.

#### Achieved power

The achieved power in this experiment was 0.91, when calculating power with the empirical effect size of *d* = 0.335 in the delayed memory test, an alpha level of 0.05 and the sample size of 82.

### Discussion

Replicating Experiment 1, the results of Experiment 2 showed the task-switching effect after 1 week. Moreover, recollection was critical for this effect, as more “remember” responses were given for repeat than for switch stimuli. In contrast, familiarity did not differ, as “know” responses did not vary with trial type.

## General discussion

The *attentional boost effect* denotes that transient increases in attention to one task can enhance memory performance in a second task (Swallow and Jiang, 2010, 2013). Here, we rather provided evidence for an *attentional attenuation effect:* When the cognitive control demands are high, the ability to attend to an item is reduced which results in lower memory performance (Reynolds et al., 2004; Craik et al., 2018; Muhmenthaler and Meier, 2019). In two experiments, we showed this effect by using a task-switching procedure at study. The results revealed a consistent memory cost for switch compared to repeat stimuli, regardless whether memory was tested immediately or after 1 week.

Interestingly, enhanced cognitive control demands do not necessarily reduce encoding capacity in all circumstances. Studies of Stroop or Flanker conflict on subsequent memory performance found improved memory performance for incongruent compared to congruent stimuli (Krebs et al., 2015; Rosner et al., 2015; Muhemnthaler and Meier, 2021a,b). In contrast, in dual-task and divided-attention situations typically a memory deficit occurs for target stimuli, similarly to the effect of task switching (Dell’Acqua and Jolicoeur, 2000; Vachon and Jolicœur, 2011; Greene and Naveh-Benjamin, 2022). Thus, there are conditions, which divert cognitive resources away from stimulus encoding, leading to lower memory and there are conditions which increase encoding of conflict stimuli (Botvinick et al., 2004). So far, these memory effects have been mainly investigated after a short study-test interval. Here we demonstrate similar consequences after a longer retention interval.

To our knowledge, there is only one task-switching study that has also investigated a longer study-test interval (Dubravac and Meier, 2022). In this study, the participants had to switch between a word and a picture classification task on compound stimuli (i.e., targets and distractors) similar to Richter and Yeung (2012). Across five experiments, memory selectivity, that is, the relative advantage of targets over distractors was tested either immediately, after one day or after 1 week. With longer retention intervals, memory selectivity washed out, but the recognition memory advantage of switch vs. repeat targets persisted. In line, our results showed a robust task-switching effect after 1 week. Compared to task switching, task repeating requires fewer working memory resources and thus provides the opportunity for more elaborated processing (Barrouillet et al., 2007; Liefooghe et al., 2008; Lavie, 2010). As elaborated processing leads to deeper memory traces, sustainable effects on learning occur, resulting in better long-term memory (Gardiner, 1988; Gardiner and Java, 1991; Bjork and Bjork, 2011; Meier and Muhmenthaler, 2021).

Our study focused on the contributions of recollection and familiarity to recognition memory performance. The results of both experiments revealed that the task-switching effect was driven by recollection. More “remember” responses were given for repeat than for switch trials. This result is in line with a recent study, in which we found the task-switching effect with a free recall test (Muhmenthaler and Meier, 2021a,b). Free recall is based on self-initiated retrieval processes which is more similar to recollection than to familiarity (Jacoby et al., 1989; Yonelinas, 2002). Thus, the present study demonstrates that recollection is at the core of the task-switching on memory effect for both free recall and recognition.

The present study also revealed that the effect of recollection was enduring. Similar results were obtained by Gardiner (1988). He investigated the long-term effects of a generation-versus-read manipulation and the contributions of recollection and familiarity. The generation effect occurs when people remember words presented as fragments better than words that are complete from the start. The effect relies on more elaborated processing in the word-generating compared to the word-reading condition (Graf, 1978; Begg et al., 1991). The results showed an enduring generation effect, and this effect was driven by recollection. Thus, as with task switching, the results showed that elaborated and effortful processing can foster long-term learning (Bjork and Bjork, 2011).

Evidence for enduring memory effects may be inferred from a neuroimaging study (Carr et al., 2010). In this study, the authors assessed memory performance for studied items both after ten minutes and after a one-week interval with the remember/know paradigm. The results showed that the encoding activity in the prefrontal cortex was significantly greater for items that later were consistently recollected (i.e., recollective in both tests) than for items which became familiar within a week or were consistently familiar. This highlights that items which are recollected later are differently processed at encoding. As enhanced prefrontal activity indicates elaborated and effortful processes, the neuropsychological data are in line with our results. An avenue for future research may be to use imaging methods to test the hypothesis that repeat items which lead to an experience of recollection at test engage more frontal activity at study.

In our study, the estimates of recollection and familiarity were assessed on a subjective level, which can be seen as a limitation. The participants were asked whether they were sure about their decisions or whether they perceived a feeling of familiarity (Tulving, 1985). In order to assess recollection on a more objective level, besides using imaging, one could also assess the retrieval of contextual detail by asking the participants in which task or on which position they had encountered a specific stimulus (*cf.*, Yonelinas and Levy, 2002). When participants can accurately respond this indicates that they have recollected some qualitative information about the encoding episode. Assessing the familiarity of the stimulus should not provide the contextual information. Assessing recollection and familiarity in this fashion might be an avenue for further research.

Finally, we want to note that the online study and the lab study resulted in very similar results, thereby giving us confidence in the validity of the results of our online experiment.

## Data availability statement

The original contributions presented in the study are included in the article/supplementary material, further inquiries can be directed to the corresponding author.

## Ethics statement

The studies involving human participants were reviewed and approved by Ethical committee of the Human faculty of the University of Bern. The participants provided their written informed consent to participate in this study.

## Author contributions

MM and BM designed the experiments and wrote the manuscript. MM analyzed the data. Both authors contributed to the article and approved the final version.

## Conflict of interest

The authors declare that the research was conducted in the absence of any commercial or financial relationships that could be construed as a potential conflict of interest.

## Publisher’s note

All claims expressed in this article are solely those of the authors and do not necessarily represent those of their affiliated organizations, or those of the publisher, the editors and the reviewers. Any product that may be evaluated in this article, or claim that may be made by its manufacturer, is not guaranteed or endorsed by the publisher.
